# Network Meta-Analysis of Calcitonin Gene-Related Peptide Receptor Antagonists for the Acute Treatment of Migraine

**DOI:** 10.3389/fphar.2019.00795

**Published:** 2019-07-12

**Authors:** Fang Xu, Wenjun Sun

**Affiliations:** Department of Encephalopathy, Third Affiliated Hospital of Beijing University of Traditional Chinese Medicine, Beijing, China

**Keywords:** calcitonin gene-related peptide, migraine, headache, telcagepant, olcegepant, rimegepant, ubrogepant

## Abstract

**Background:** Research has indicated that calcitonin gene-related peptide (CGRP) receptor antagonists can be effective in the acute treatment of migraine. Six major drugs are included within this category: telcagepant, olcegepant, BI 44370, rimegepant (BMS-927711), MK3207, and ubrogepant. However, no previous studies have performed network meta-analyses to directly compare the effects of these drugs. In the present study, we assessed the therapeutic qualities of these six different drugs to inform further clinical research.

**Methods:** We searched PubMed, Embase, Ovid MEDLINE, Web of Science, and the Cochrane Central Register for Controlled Trials for relevant randomized controlled trials (RCTs) published through to October 2018. Two reviewers performed a network meta-analysis of efficacy and toxicity on the basis of odds ratios (ORs).

**Results:** Ten randomized controlled trials involving 8,174 patients were included in our analysis. Olcegepant (OR: 4.09; CI: 1.81, 9.25), ubrogepant (OR: 2.11; CI: 1.10, 4.05), and BI 44370 (OR: 3.36; CI: 2.24, 5.04) were more effective in ensuring pain relief 2 h after treatment than was placebo treatment. BI 44370 was associated with an increased risk of adverse events when compared with placebo treatment (OR: 1.57; CI: 1.32, 1.88). Surface under the cumulative ranking curve analysis revealed that olcegepant was most effective and ubrogepant was associated with the lowest risk of adverse events among the six treatment options.

**Conclusion:** Olcegepant was more effective, and ubrogepant had lower toxicity than the remaining treatments. CGRP antagonists are promising for the acute treatment of migraine, especially among patients who are unable to take triptans.

## Introduction

Migraine is the third most prevalent and second most disabling neurological disease (Geneva, 2001; GBD, 2016; GBD, 2017), with a lifetime prevalence of 33% in women and 13% in men (Younger, 2016). Occurring most frequently between the ages of 25 and 50 years, migraine is associated with a series of neurological and systemic symptoms. Indeed, the condition is characterized by recurrent moderate-to-severe headache accompanied by photophobia, phonophobia, cutaneous allodynia, and nausea. The headache attacks last from 4 to 72 h, and the average frequency of attacks is typically one or two times per month (IHS, 2018). Approximately 11% of adults worldwide experience migraine attacks, which significantly impact both quality of life and productivity (Chan et al., 2011). Currently, acute migraine treatments are largely abortive in nature. Migraine is primarily treated using triptans and/or nonsteroidal anti-inflammatory drugs (NSAIDs), although central analgesics may be used in some cases. However, when binding to effective receptors, triptans may also cause vasoconstriction of the cranial blood vessels and are thus contraindicated in patients with vascular diseases (Silberstein, 2004; Martelletti, 2015). Furthermore, because NSAIDs exert nonspecific anti-inflammatory effects that can alter gastrointestinal and renal function, they are not indicated for long-term use.

Calcitonin gene-related peptide (CGRP) is a member of the calcitonin family of peptides that is produced in both peripheral and central neurons. After CGRP is generated, it is captured by CGRP receptor and forms a complex. Also, the receptor complex functions by the G protein-coupled receptor calcitonin receptor-like receptor, and receptor activity-modifying protein 1 to mediate signal transduction and active adenylyl cyclise and cyclic adenosine monophosphate-dependent pathways, which results in level change of cyclic adenosine monophosphate active protein kinase A and phosphorylation of several downstream targets, dilate the blood vessel and helping to transmit pain signals (McLatchie et al., 1998; Mallee et al., 2002; Hay and Pioszak, 2016). Previous research has also indicated that levels of CGRP increase during migraine attacks (Goadsby and Edvinsson, 1993; Tvedskov et al., 2005). Given the role of CGRP in migraine, several research groups have aimed to determine the clinical potential of CGRP receptor antagonists. However, research related to several such agents has been halted because of concerns regarding hepatotoxicity. Therefore, in the present study, we aimed to review the potential value of several CGRP receptor antagonists in the acute treatment of migraine.

## Methods

### Literature Search Strategy

We systematically searched electronic databases, including PubMed, Embase, Ovid MEDLINE, Web of Science, and the Cochrane Central Register of Controlled Trials, for relevant studies published in English through October 2018. The following search terms were used: acute treatment, migraine, headache, calcitonin gene-related peptide, calcitonin gene-related peptide receptor antagonists, CGRP-receptorantagonists, telcagepant, olcegepant, BI 44370, rimegepant (BMS-927711), MK3207, ubrogepant, and randomized clinical trials.

The reference lists of included articles were also manually reviewed for other potentially eligible trials. Preferred Reporting Items for Systematic Reviews and Meta-Analyses (PRISMA) guidelines and the PRISMA Extension were used for reference.

### Study Eligibility

The following inclusion criteria were used to determine study eligibility: 1) Randomized controlled trials (RCTs) involving adult patients with migraine, patients’ number with at least 10; 2) acute treatment with CGRP receptor antagonists, with at least one reported outcome measure [pain-free at 2 h, adverse events (AEs), and drug-related AEs]; 3) publication in English; 4) inclusion of a placebo or control arm; 5) with interesting results. When there were multiple publications associated with a single trial, we included only the most recent version. Updated data were regarded as new evidence. Two investigators (FX and WS) independently determined whether each study met the inclusion criteria, and all disagreements were resolved *via* consensus.

### Data Extraction and Outcome Measures

Basic information was extracted by two investigators (FX and WS). Two-hour pain-free rate was regarded as the primary outcome measure. The toxicity and tolerability of each treatment were assessed based on AEs and drug-related AEs.

### Risk of Bias

The risk of bias for each study was evaluated using the Cochrane risk of bias tool (Higgins et al., 2011). This tool allows for the assessment of sequence generation, allocation concealment, blinding, incomplete outcome data, and selective reporting for each RCT. Included RCTs were categorized as follows based on the risk of bias: low, high, or unclear.

### Statistical Analysis

Network meta-analysis (NMA) is a method that combines direct and indirect evidence and synthesis data obtained *via* direct or indirect comparisons of different treatments and allow for inferences regarding the comparative effects of interventions without direct comparators. NMA can thus provide better comparative evidence than pair-wise meta-analysis. Because no previous RCTs have directly compared the effects of different CGRP receptor antagonists among patients with migraine, we adopted a Bayesian framework using the Markov chain Monte Carlo method. The network was constructed by comparing the following six major drugs: telcagepant, olcegepant, BI 44370, rimegepant (BMS-927711), MK3207, and ubrogepant. The comparative efficacy and toxicity of each treatment (i.e., 2-h pain-free rate and AE rate) was summarized using odds ratios (ORs) and the corresponding 95% credible intervals, the Bayesian equivalent of 95% confidence intervals (CIs). The inconsistency of the NMA was evaluated to determine the conformity between direct and indirect sources of evidence. The NMA yielded a ranking probability curve, which estimates the probability of each treatment to achieve the best rank among all treatments. When a treatment is certain to be the best, the surface under the cumulative ranking (SUCRA) value equals one (or 100%). Conversely, when a treatment is certain to be the worst, the SUCRA line equals zero (or 0%) (Salanti et al., 2011; Chaimani et al., 2013).

All statistical tests were two-sided; *P* ≤ 0.05 was with statistical significance.

Statistical analyses were performed using the mvmeta command in Stata version 11.2 (StataCorp, College Station, TX, USA) (Chaimani et al., 2013). Mvmeta conducts meta-analysis of random multivariate effects and meta-regression of random multivariate effects on a data set of estimates, variances, and (optionally) covariances. The risk of bias was assessed using the Cochrane tool in Review Manager (RevMan, version 5.3, The Nordic Cochrane Centre, The Cochrane Collaboration, Copenhagen, Denmark).

## Results

**Figure 1:**
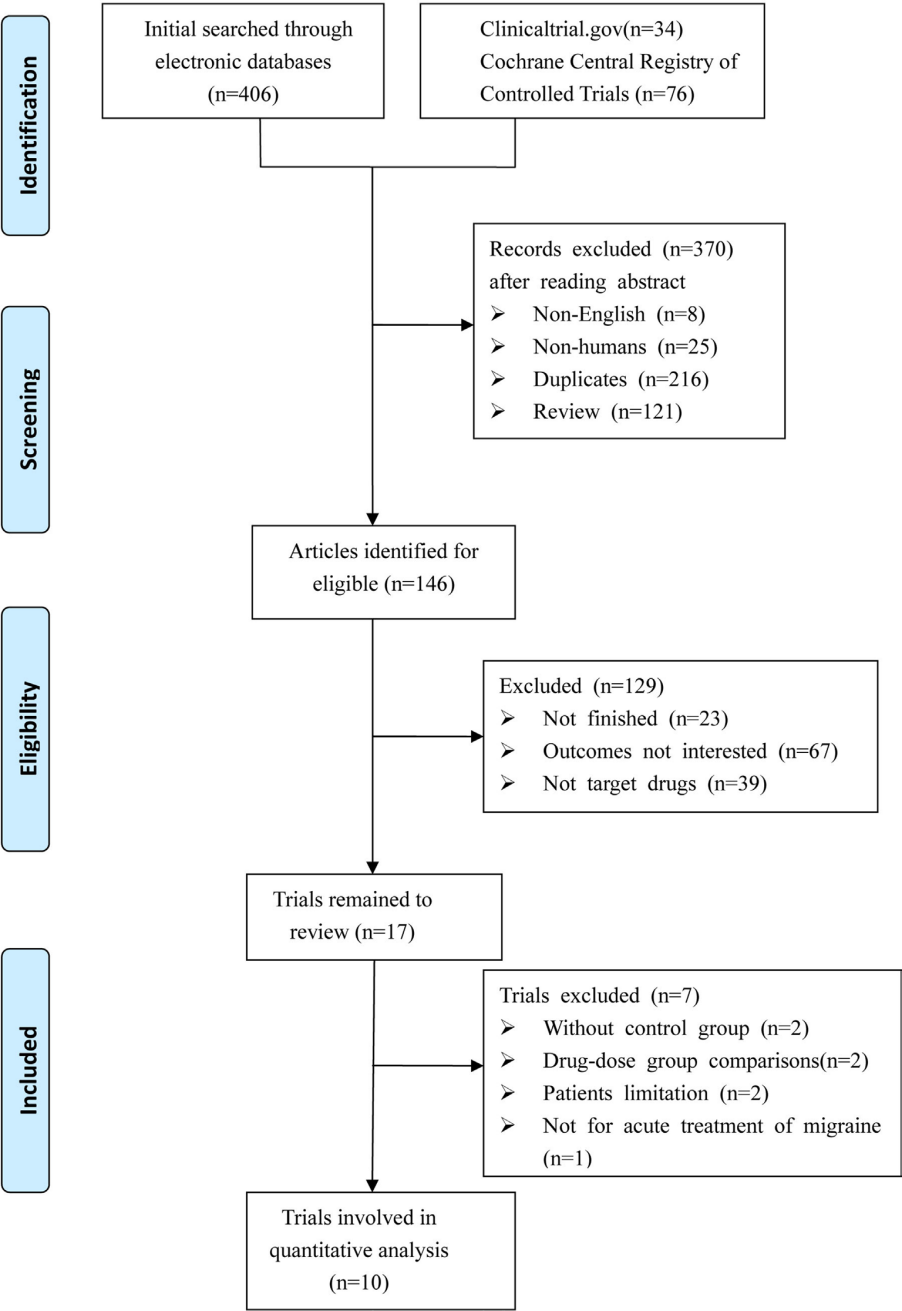
Diagram of eligible studies selection procedures.

We identified a total of 516 relevant references based on a review of titles and abstracts. After excluding 216 duplicates and 283 articles not meeting the inclusion criteria (eight with non-English; 25 were not human researches; 121 were reviews; 23 were not finished; 67 with no interesting results; 39 were not target drugs), 17 trials remained. Among these, two were excluded due to the lack of control group, two were excluded due to study with no appropriate control group, two were excluded due to patients limited to certain conditions (e.g. coronary artery disease), and one was excluded due to a focus on migraine prevention. Thus, 10 RCTs met the eligibility criteria for our study. A total of 8,174 patients were included in the NMA. **Figure 1** shows the procedures for the literature search and selection of clinical trials. The characteristics of the 10 included trials are summarized in **Table 1** (Olesen et al., 2004; Ho et al., 2007; Ho et al., 2008; Connor et al., 2009; Ho et al., 2010; Connor et al., 2011; Diener et al., 2011; Hewitt et al., 2011; Marcus et al., 2014; Voss et al., 2016).

**Table 1:** Baseline characteristics of involved patients.

First author year	Drugs		No. of patients		Age		Male sex		Prior therapy								Aura		Moderate–severe headache	
									Type											
									NSAIDs		Triptan		NSAIDs + Triptan		Other					
	T	C	T	C	T	C	T	C	T	C	T	C	T	C	T	C	T	C	T	C
(Olesen et al., 2004)	Olcegepant	Placebo	85	41	47	47	14	12	NA	NA	NA	NA	NA	NA	NA	NA	10	5	4	2
(Ho et al., 2007)	Telcagepant	Placebo	181	115	41.2	42.2	21	11	61	40	70	35	23	24	26	13	45	13	181	115
	Telcagepant	Rizatriptan	181	34	41.2	40.2	21	6	61	8	70	16	23	4	26	5	45	8	181	34
(Ho et al., 2008)	Telcagepant	Placebo	687	348	42.6	42.3	110	54	166	99	312	148	129	66	63	31	114	67	685	345
	Telcagepant	Zolmitriptan	687	345	42.6	41.7	110	47	166	86	312	154	129	63	63	34	114	63	685	343
(Connor et al., 2009)	Telcagepant	Placebo	929	365	41.6	41.9	124	47	254	96	395	166	152	56	117	39	222	82	365	929
(Ho et al., 2010)	Telcagepant	Placebo	1122	555	43	42.5	161	92	289	128	490	240	203	110	117	66	164	89	1,117	551
(Diener et al., 2011)	BI 44370 TA	Placebo	202	70	41.6	38.2	40	9	NA	NA	NA	NA	NA	NA	NA	NA	NA	NA	199	70
	BI 44370 TA	Eletriptan	202	69	41.6	37.9	40	8	NA	NA	NA	NA	NA	NA	NA	NA	NA	NA	199	68
(Connor et al., 2011)	Telcagepant	Rizatriptan	641	313	42.5	41.9	139	76	219	96	253	117	98	62	57	29	NA	NA	641	313
(Hewitt et al., 2011)	MK-3207	Placebo	407	140	42.8	42.1	56	15	NA	NA	326	117	NA	NA	NA	NA	NA	30	407	140
(Marcus et al., 2014)	Rimegepant	Placebo	547	229	39.6	37.9	98	33	NA	NA	NA	NA	NA	NA	NA	NA	NA	NA	547	229
	Rimegepant	Sumatriptan	547	109	39.6	40.6	98	18	NA	NA	NA	NA	NA	NA	NA	NA	NA	NA	547	109
(Voss et al., 2016)	Ubrogepant	Placebo	527	113	40.9	40.5	67	15	376	82	216	44	NA	NA	111	26	NA	NA	527	113

NA, not available; T, treatment group; C, control group; NSAIDs, nonsteroidal anti-inflammatory drugs; NO, number.

Four trials included three arms involving 2,936 patients, while the others included two arms involving 5,238 patients. Four trials were in phase III, while the remaining six were in phase II. Five trials investigated telcagepant treatment, while olcegepant, BI 44370, MK-3207, rimegepant, and ubrogepant treatments were investigated in one trial each. The strategies adopted in each study are presented in **Table 2**.

**Table 2:** Characteristics of trials.

Study	Phase	Regimens		Blinding
		Treatment group	Control group	
(Olesen et al., 2004)	2	0.1, 0.25, 0.5, 1, 2.5, 5, 10 mg Olcegepant	Placebo	Double-blind
(Ho et al., 2007)	2	25, 50, 100, 200, 300, 400, 600 mg Telcagepant (MK-0974)	10 mg Rizatriptan or placebo	Double-blind
(Ho et al., 2008)	3	150 mg, 300 mg Telcagepant	5 mg Zolmitriptan or placebo	Double-blind
(Connor et al., 2009)	2	50, 150, 300 mg Telcagepant	Placebo	Double-blind
(Ho et al., 2010)	3	140 mg, 280 mg Telcagepant	Placebo	Double-blind
(Connor et al., 2011)	3	300 mg, 280 mg Telcagepant	10 mg Rizatriptan	Double-blind
(Diener et al., 2011)	2	50, 200, 400 mg BI 44370 TA	40 mg Eletriptan or placebo	Double-blind
(Hewitt et al., 2011)	2	2.5, 5, 10, 20, 50, 100, 200 mg MK-3207	Placebo	Double-blind
(Marcus et al., 2014)	2	10, 25, 75, 150, 300, 600 mg Rimegepant (BMS-927711)	100 mg Sumatriptan or placebo	Double-blind
(Voss et al., 2016)	2	1, 10, 25, 50, 100 mg Ubrogepant	Placebo	Double-blind

### Efficacy

**Figure 2:**
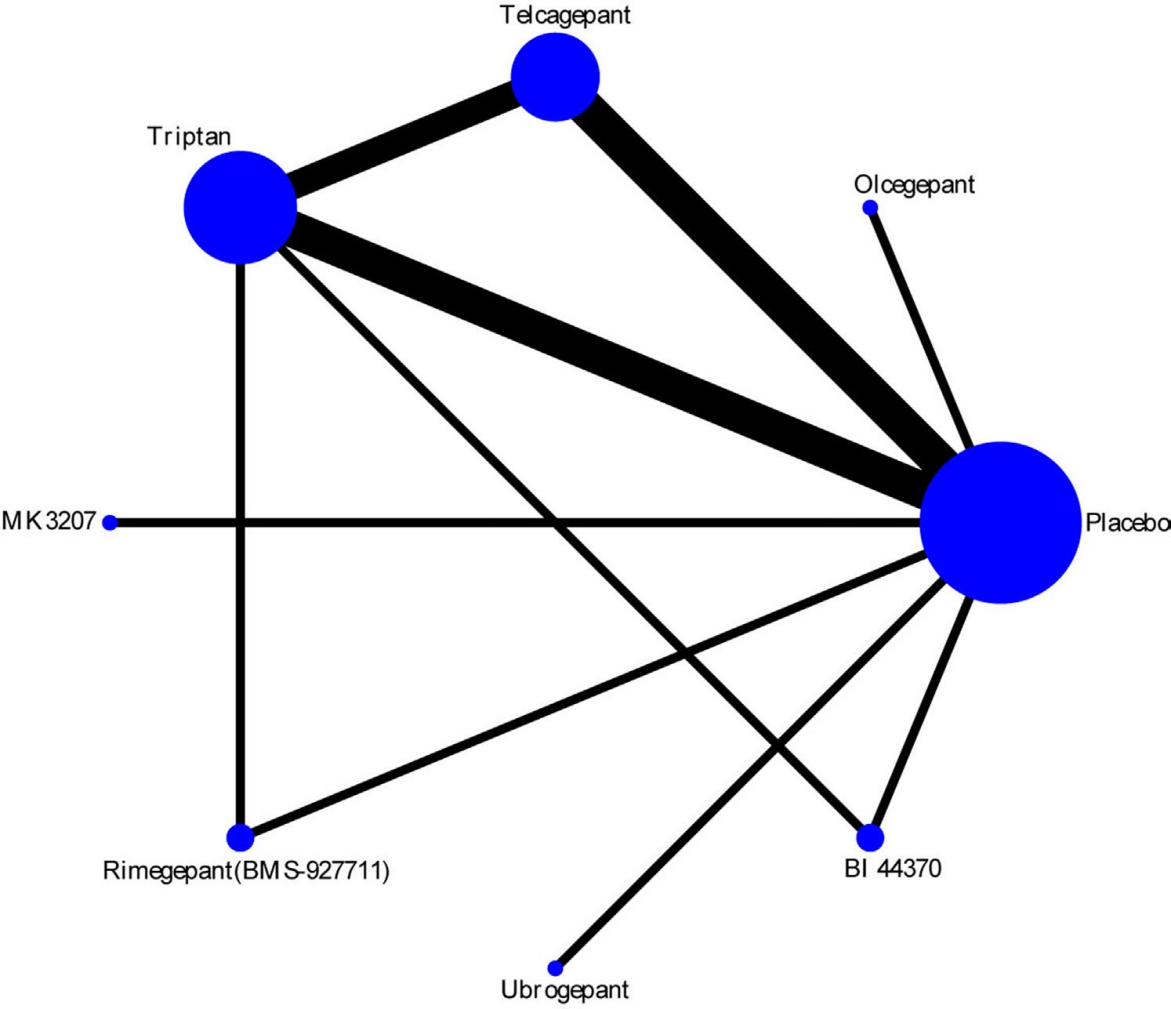
Network plot for 2-h pain-free of six different calcitonin gene-related peptide (CGRP) antagonists. Lines represent direct comparisons within the randomized controlled trials (RCTs). The line thickness indicates the number of RCTs included in each comparison.

**Figure 3:**
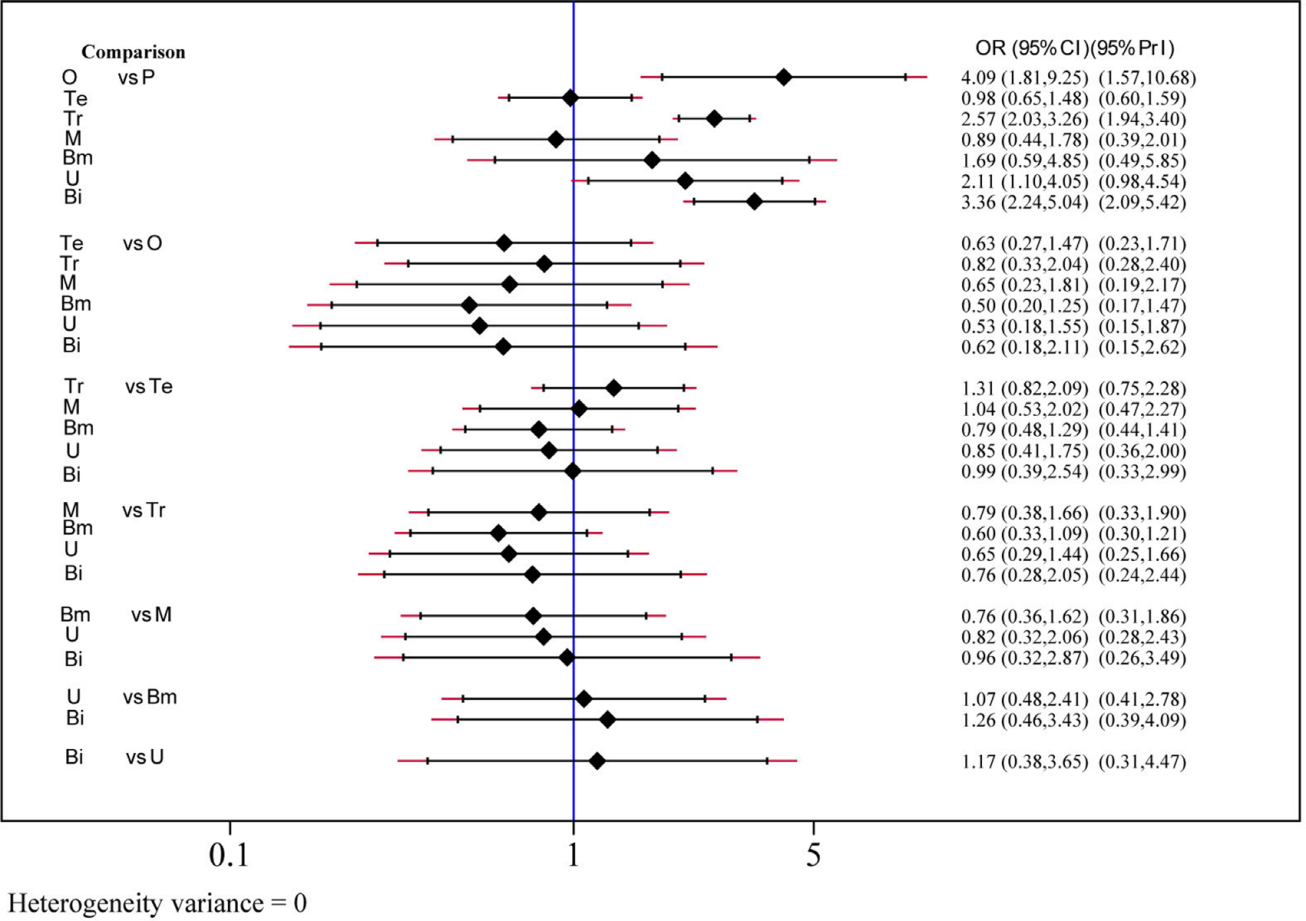
Efficacy analysis results for 2-h pain-free of six treatment modalities. OR, odds ratio; CI, confidence interval; PrI, predictive interval; O, olcegepant; Te, telcagepant; Tr, triptan; M, MK3207; Bm, rimegepant (BMS-927711); U, ubrogepant; Bi, BI 44370; P, placebo.

The efficacy of each drug was evaluated on the basis of the 2-h pain-free rate. Nine comparisons across 10 trials were included in network plot (**Figure 2**). The NMA revealed that olcegepant (OR: 4.09; CI: 1.81, 9.25), ubrogepant (OR: 2.11; CI: 1.10, 4.05), and BI 44370 (OR: 3.36; CI: 2.24, 5.04) were more effective than placebo treatment. Furthermore, olcegepant treatment was superior to the other five treatments, although these differences were not statistically significant (**Figure 3**). Our results also indicated that triptans were marginally less effective than olcegepant treatment, based on the 2-h pain-free rate (OR: 0.82; CI: 0.33, 2.04). SUCRA analysis indicated that olcegepant was most likely to be the best treatment (SUCRA: 0.84; PrBest: 53.3%), followed by triptans (SUCRA: 0.78; PrBest: 20.7%) and MK-3207 (SUCRA: 0.55; PrBest: 4.1%) (**Table S1**).

### Safety and Toxicity

**Figure 4:**
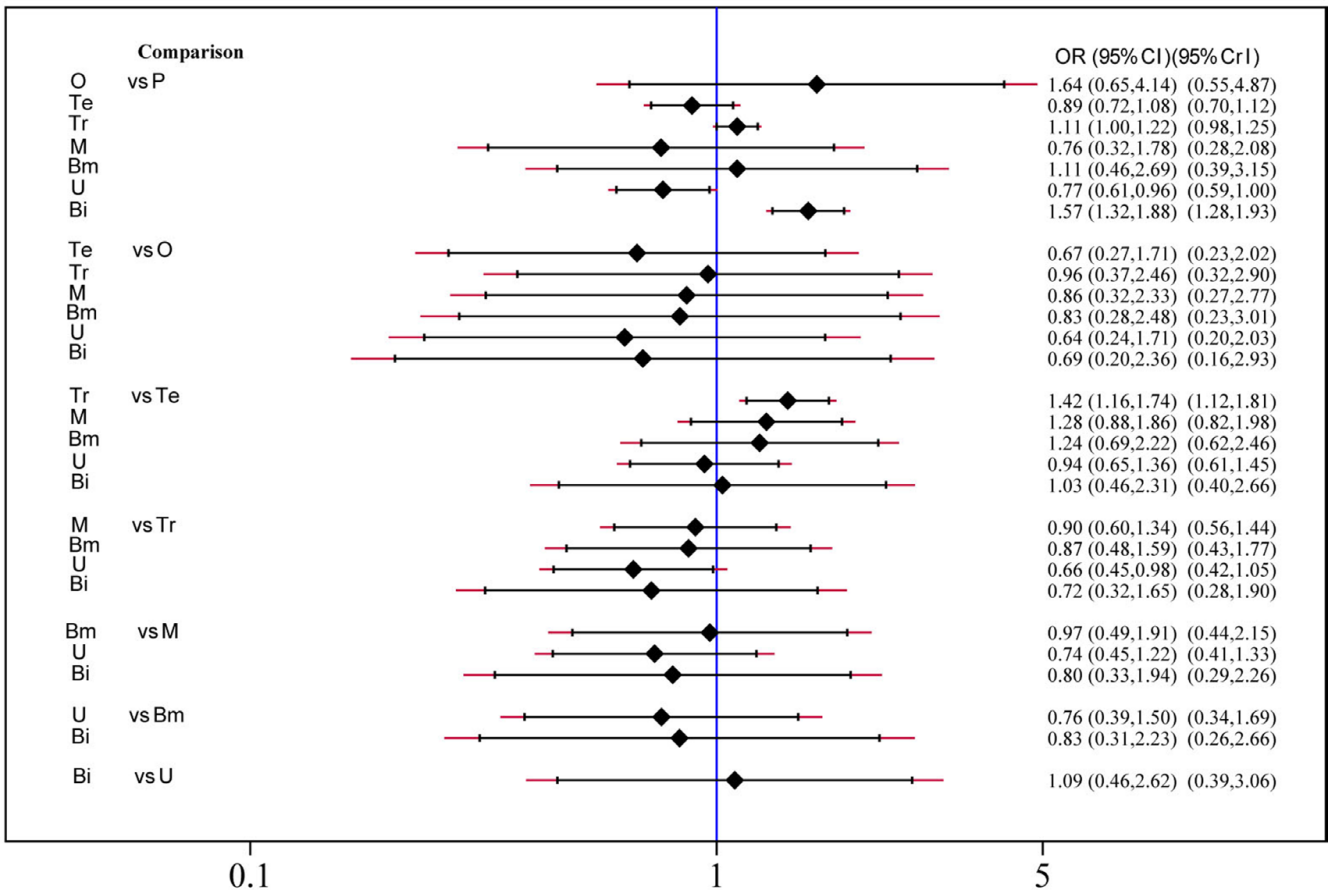
Adverse events analysis of six treatment modalities. OR, odds ratio; CI, confidence interval; PrI, predictive interval; O, olcegepant; Te, telcagepant; Tr, triptan; M, MK3207; Bm, rimegepant (BMS-927711); U, ubrogepant; Bi, BI 44370; P, placebo.

**Figure 5:**
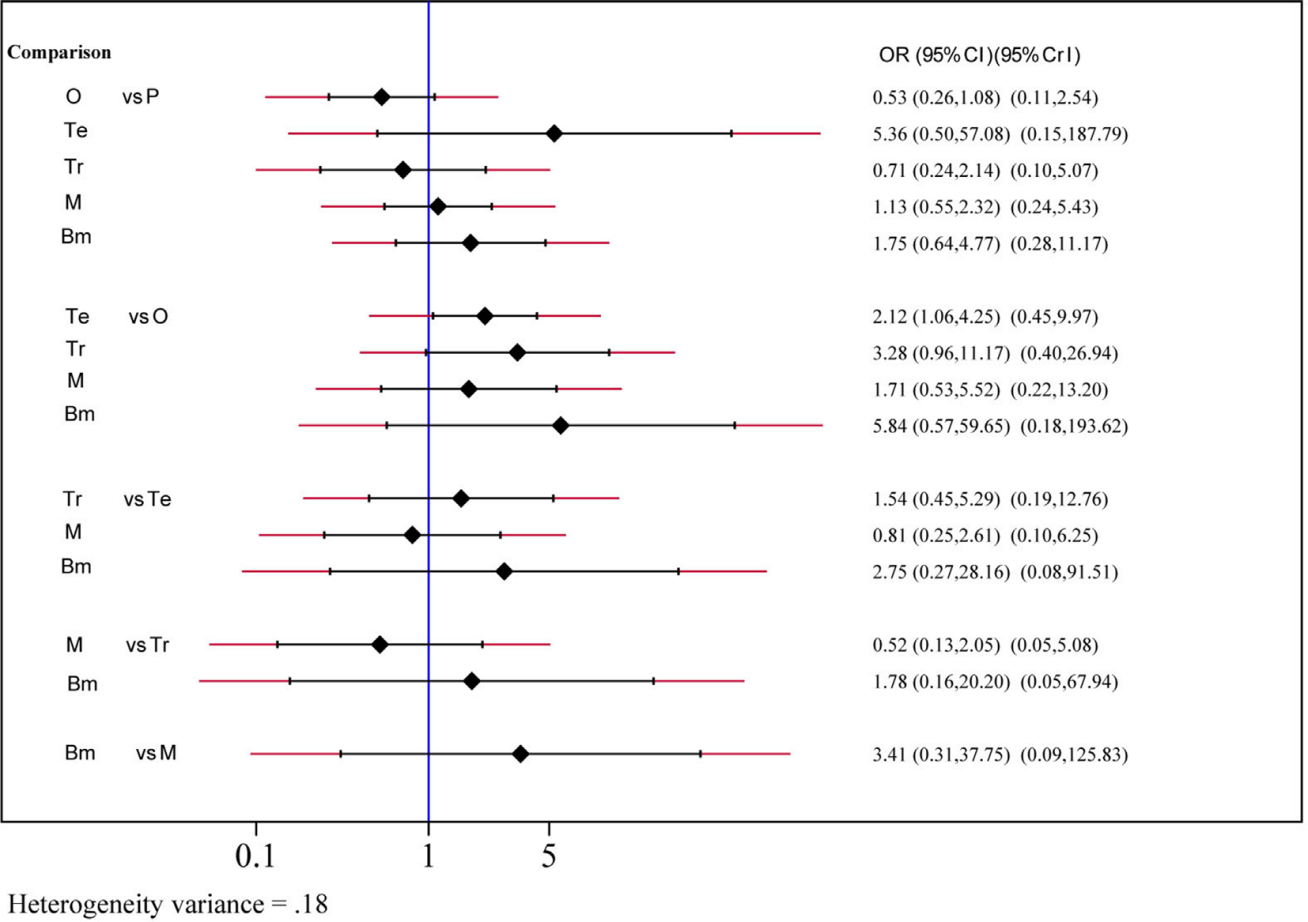
Drug-related adverse events analysis of six treatment modalities. OR, odds ratio; CI, confidence interval; PrI, predictive interval; O, olcegepant; Te, telcagepant; Tr, triptan; M, MK3207; Bm, rimegepant (BMS-927711); P, placebo.

Safety and toxicity were evaluated on the basis of AEs and drug-related AEs. There were no significant differences in AEs among telcagepant, olcegepant, MK-3207, and rimegepant. BI 44370 was associated with an increased risk of AEs (OR: 1.57; CI: 1.32, 1.88), and ubrogepant was even with lower risk of AEs (OR: 0.77; CI: 0.61, 0.96) when compared with placebo treatment (**Figure 4**). The SUCRA analysis suggested that ubrogepant was associated with the lowest risk of AEs (SUCRA: 0.73) (**Table S1**). There were no significant differences in drug-related AEs among the six treatments (**Figure 5**).

### Rank Analysis

**Figure 6:**
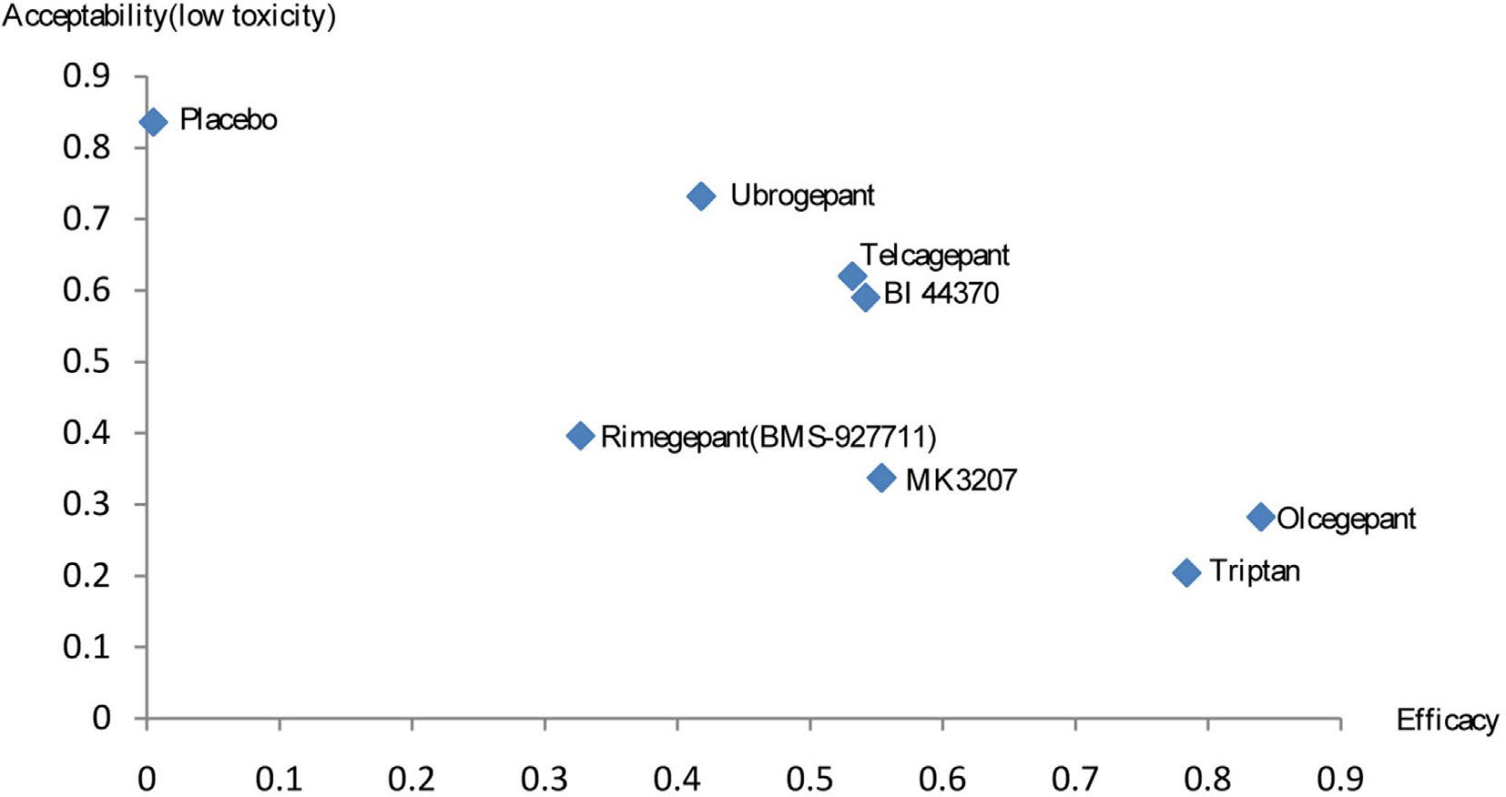
Rank results for efficacy and acceptability (low toxicity).

A combined ranking plot was used to evaluate both efficacy (2-h pain-free rate) and toxicity (AEs) (**Figure 6**). The ideal treatment option should appear in the upper right corner (i.e., higher efficacy and acceptability, acceptability is equal to low toxicity). Our analysis indicated that olcegepant was more effective than the remaining treatments, including triptans. Although toxicity values were lower for the other five treatments than for olcegepant, these treatments were associated with lower efficacy.

### Assessment of Heterogeneity and Inconsistency

There was no heterogeneity in the NMA for 2-h pain-free rate and AEs (tau-square = 0, *I* ^2^ = 0.0%). However, drug-related AEs (tau-square = 0.105, *I* ^2^ = 55.5%) exhibited heterogeneity, as shown in **Supplementary Table S1**. Although inconsistency was observed in the meta-analysis of AEs (*P* = 0.02), it was not observed in the networks for other indices (**Table S2**). We did not perform meta-regression analyses to explore sources of inconsistency due to the limited amount of existing data for direct treatment comparisons.

### Risk of Bias

We examined all articles using the Cochrane risk of bias tool. Our analysis indicated that most trials were of low risk, as shown in **Figures S1** and **S2**.

## Discussion

CGRP is a potent neurotransmitter in the central nervous system, and recent studies have highlighted the potential of CGRP receptor blockade in the acute treatment of migraine. However, most studies have only investigated the clinical efficacy of individual agents relative to placebo treatment, rather than the efficacy of different CGRP antagonists in relation to one another. Thus, in the present study, we examined the efficacy and toxicity of six major CGRP antagonists.

Among the six major treatments investigated in our NMA (olcegepant, MK3207, BI 44370, telcagepant, ubrogepant, and rimegepant), olcegepant exhibited the greatest efficacy, based on the 2-h pain-free rate in patients with migraine. Furthermore, olcegepant was superior to triptans in the rank analysis, which considered both efficacy and toxicity. We also observed that MK3207 and rimegepant were associated with moderate efficacy and moderate toxicity, while ubrogepant, telcagepant, and BI 44370 were associated with moderate efficacy and low toxicity. In addition, our results indicated that olcegepant, MK3207, BI 44370, telcagepant, ubrogepant, and rimegepant were associated with lower toxicity than triptans. Previous meta-analysis study evaluated the efficacy of CGRP antagonists in acute treatment of migraine compared with that of triptans (Hong and Liu, 2017), and CGRP antagonists was as one class of drug. In current study, we separated CGRP antagonists as individual comparisons (e.g. olcegepant, MK3207, BI44370, telcagepant, ubrogepant, and rimegepant). This enabled us to analyze the effect of each kind of CGRP antagonists for the acute treatment of migraine.

There several advantages in this study. It is the first NMA to compare different CGRP antagonists in the acute treatment of migraine, analyzing the effect of single drug in treatment and providing new clinical insight. Secondly, in rank analysis, we integrate the data of drug effects and adverse rates together for making assessments thoroughly, meanwhile, make the illustration within one graph to interpret clearly.

Although our results indicated that olcegepant was the most effective of the six treatments investigated, research regarding olcegepant has been discontinued due to its high molecular weight and limited ability to penetrate the brain (Gonzalez-Hernandez et al., 2018; Holland and Goadsby, 2018). In addition, although telcagepant and BI 44370 were associated with moderate efficacy and low toxicity in acute intermittent treatment, research regarding these compounds has been discontinued due to hepatotoxicity concerns during long-term prophylactic use (Connor et al., 2011; Diener et al., 2011). However, prolonged intermittent use of telcagepant results in only transient increases in liver aminotransferase, and there is no evidence to suggest that telcagepant alters liver function (Ho et al., 2014; Ho et al., 2016). Research regarding MK3207, which exhibited moderate efficacy and moderate toxicity in our study, has also been suspended due to concerns regarding hepatotoxicity. However, ongoing studies are investigating the clinical potential of ubrogepant (moderate efficacy, low toxicity) and rimegepant (moderate efficacy, moderate toxicity), the latter of which exerts minimal effects on liver transaminases.

Triptans represent the first-line treatment for acute migraine. However, because triptans bind both the receptors located on peripheral trigeminal sensory nerve endings and those located on intracranial, extracranial, and systemic blood vessels (Dodick et al., 2004; Marmura et al., 2015), the mechanisms underlying their efficacy can also cause vasoconstriction. Thus, triptans are not suitable for patients with vascular risk factors. Moreover, research has indicated that approximately 30 to 40% of patients do not respond adequately to triptan therapy (Viana et al., 2013). Thus, it is necessary to identify alternative treatment strategies for patients with migraine. CGRP antagonists have been promising in recent studies, especially with regard to AEs, as these drugs exhibit toxicity levels lower than or similar to those of triptans. Studying these drugs, whether discontinued or not, is of utmost importance for current and future drug discovery.

### Limitations

Our study has several limitations to note. First, although all eligible trials had similar inclusion criteria, some differences were unavoidable, such as slight differences in patient characteristics (e.g., the presence or absence of aura, variations in previous drug treatment, etc.). Although these factors may have influenced our results, we did not perform subgroup analyses because of a lack of relevant information. Second, as research regarding some drugs has been suspended, data on olcegepant, MK3207, BI 44370, ubrogepant, and rimegepant were limited. Given that only one trial investigated each of these agents, our results should be interpreted with caution.

## Conclusion

Olcegepant was more effective and ubrogepant had lower toxicity than the remaining treatments. CGRP antagonists are promising for the acute treatment of migraine, especially among patients who cannot take triptans or who have not responded to triptan treatment. Further studies regarding ubrogepant and rimegepant may dispel previous concerns regarding the hepatotoxicity of CGRP antagonists.

## Author Contributions

Conception of the study: WS; literature search: WS and FX; data extraction: FX and WS; statistical analysis: FX; drafting the manuscript: FX; revising and completion of final work: WS; all authors reviewed and approved the final manuscript.

## Funding

The Project of Beijing Municipal Science and Technology Commission (no. Z151100004015139) and Xin'ao funding project of Beijing University of Chinese Medicine (no.2017-XAJLJJ-021).

## Conflict of Interest Statement

The authors declare that the research was conducted in the absence of any commercial or financial relationships that could be construed as a potential conflict of interest.

## Abbreviations

AEs, adverse events; CGRP, calcitonin gene-related peptide; CIs, confidence intervals; NMA, network meta-analysis; NSAIDs, nonsteroidal anti-inflammatory drugs; ORs, odds ratios; PRISMA, Preferred Reporting Items for Systematic Reviews and Meta-Analyses; RCTs, randomized controlled trials; SUCRA, surface under the cumulative ranking curve.

## Supplementary Material

Figure S1 Risk of bias summary for each risk of bias item for each included study.

Figure S2 Risk of bias graph for each risk of bias item presented as percentages across all included studies.

Table S1 Rank results.

Table S2 Assessment of heterogeneity and inconsistency.
