# Supplementary figures and images for: Network Meta-Analysis of Calcitonin Gene-Related Peptide Receptor Antagonists for the Acute Treatment of Migraine
Source: Front Pharmacol. 2019 Jul 12;10:795. doi: 10.3389/fphar.2019.00795 (PMC6640487; doi:10.3389/fphar.2019.00795)

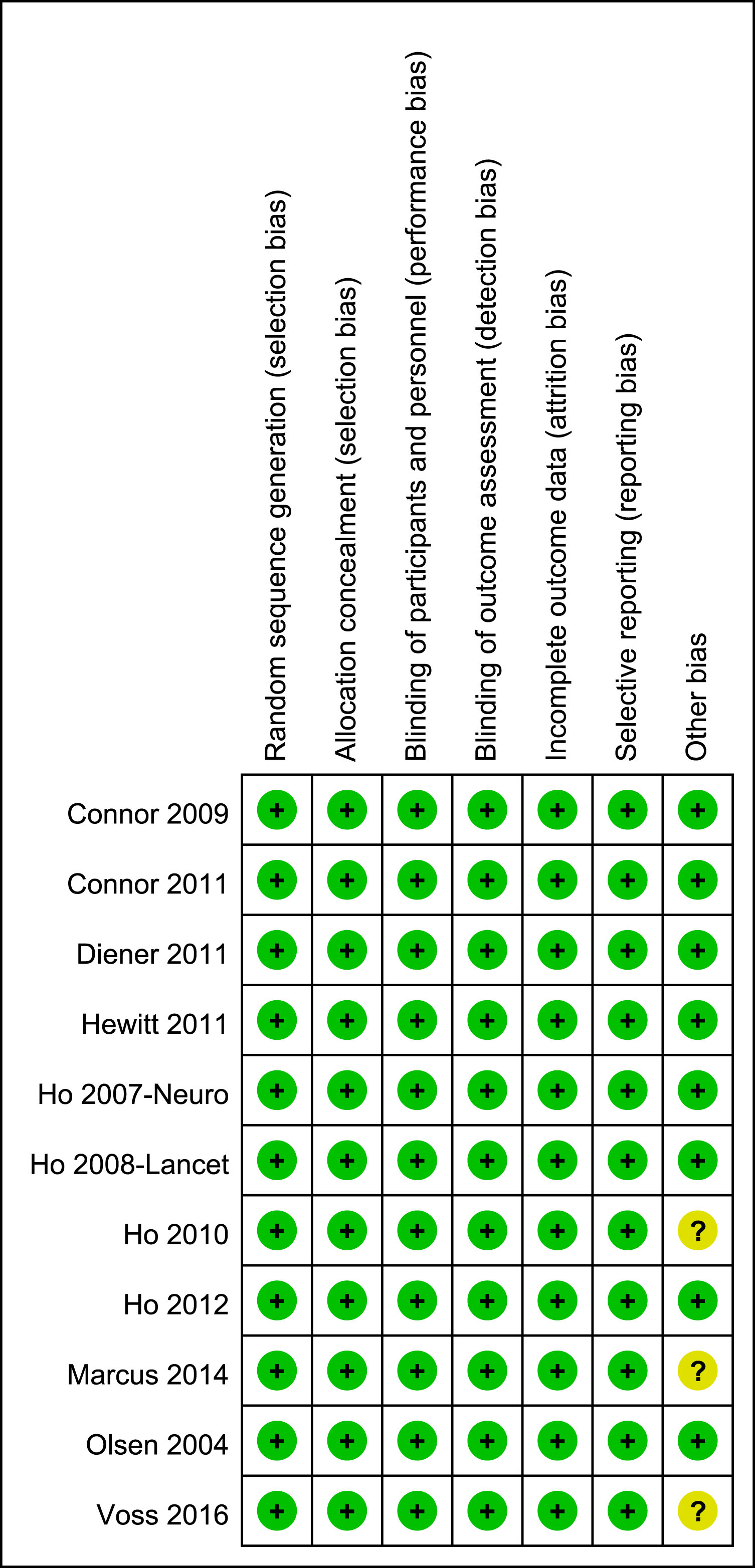

Supplement: Figure S1 — Risk of bias summary for each risk of bias item for each included study. [file Image_1.jpeg]

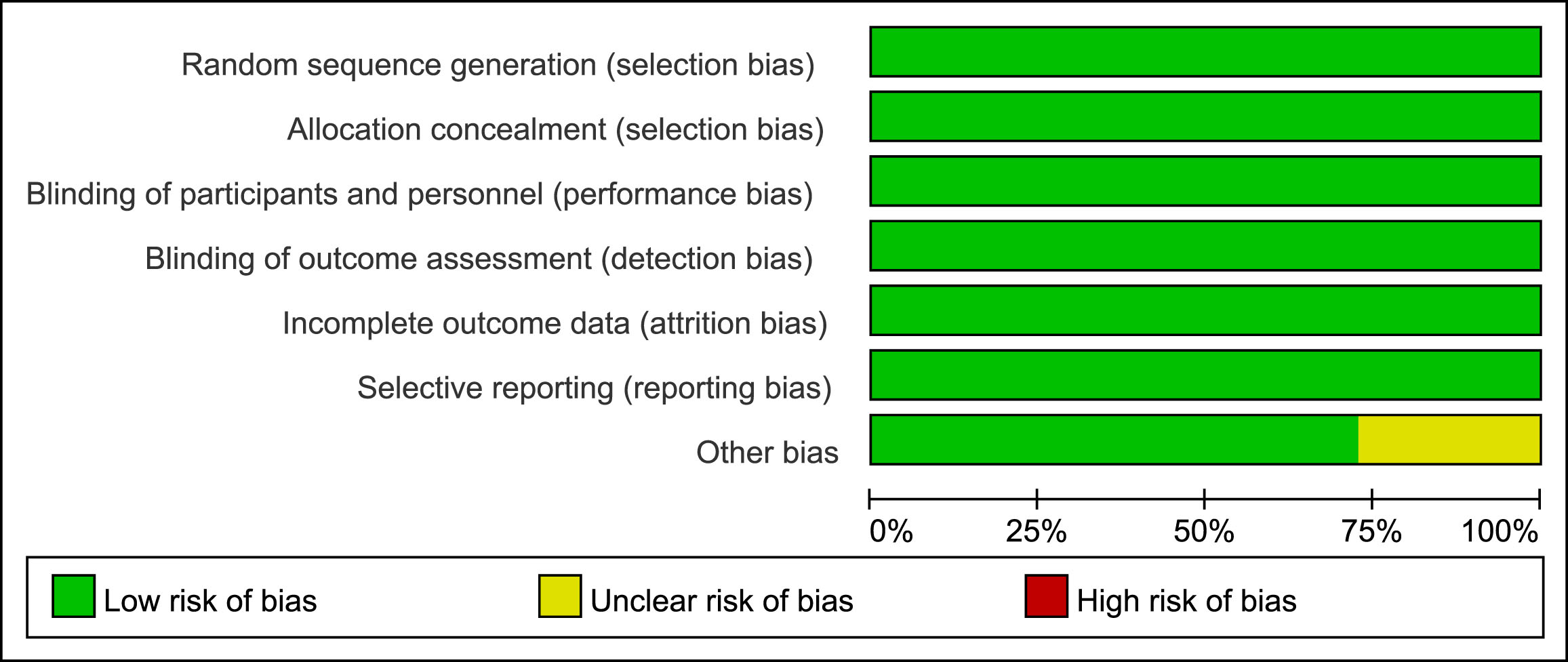

Supplement: Figure S2 — Risk of bias graph for each risk of bias item presented as percentages across all included studies. [file Image_2.jpeg]
