# Supplementary material for: Network Meta-Analysis of Calcitonin Gene-Related Peptide Receptor Antagonists for the Acute Treatment of Migraine
Source: Front Pharmacol. 2019 Jul 12;10:795. doi: 10.3389/fphar.2019.00795 (PMC6640487; doi:10.3389/fphar.2019.00795)
Supplement: Table S1 — Rank results. [file Table_1.docx]

|  | Treatments | SUCRA | PreBest (%) | MeanRank |
| --- | --- | --- | --- | --- |
| 2-h-pain-free | placebo | 0.005 | 0.0 | 8.0 |
|  | olcegepant | 0.838 | 53.3 | 2.1 |
|  | telcagepant | 0.532 | 1.1 | 4.3 |
|  | triptan | 0.784 | 20.7 | 2.5 |
|  | MK3207 | 0.554 | 6.2 | 4.1 |
|  | rimegepant (BMS-927711) | 0.327 | 0.3 | 5.7 |
|  | ubrogepant | 0.418 | 4.3 | 5.1 |
|  | BI 44370 | 0.542 | 14.1 | 4.2 |
|  |  |  |  |  |
| Acceptability  (low adverse events) | placebo | 0.836 | 24.7 | 2.1 |
|  | olcegepant | 0.282 | 9.0 | 6.0 |
|  | telcagepant | 0.623 | 0.6 | 3.6 |
|  | triptan | 0.204 | 0.0 | 6.6 |
|  | MK3207 | 0.337 | 1.5 | 5.6 |
|  | rimegepant (BMS-927711) | 0.396 | 7.7 | 5.2 |
|  | ubrogepant | 0.732 | 25.4 | 2.9 |
|  | BI 44370 | 0.59 | 31.1 | 3.9 |

**Table S1.Rank results**

PreBest: prediction of best
