# Supplementary material for: Network Meta-Analysis of Calcitonin Gene-Related Peptide Receptor Antagonists for the Acute Treatment of Migraine
Source: Front Pharmacol. 2019 Jul 12;10:795. doi: 10.3389/fphar.2019.00795 (PMC6640487; doi:10.3389/fphar.2019.00795)
Supplement: Table S2 — Assessment of heterogeneity and inconsistency. [file Table_2.docx]

Table S2. Testing for inconsistency and heterogeneity

|  | 2H pain-free | Adverse | Drug-related adverse |
| --- | --- | --- | --- |
| Inconsistency | _trtdiff_Tr_des_BmPTr = 0  _trtdiff_Tr_des_PTeTr = 0  _trtdiff_Te_des_PTeTr = 0  _trtdiff_Te_des_TeTr = 0 | _trtdiff_Tr_des_BmPTr = 0  _trtdiff_Tr_des_PTeTr = 0  _trtdiff_Te_des_PTeTr = 0  _trtdiff_Te_des_TeTr = 0 | _trtdiff_Tr_des_PTeTr = 0  _trtdiff_Te_des_PTeTr = 0  _trtdiff_Te_des_TeTr = 0 |
|  | F( 4, 8) = 3.02 | F(4, 8) = 5.52 | F( 3,5) = 2.34 |
|  | P= 0.09 | P =0.02 | P = 0.20 |
| Heterogeneity | tau2 = 0 | tau2 = 0 | tau2 = 0.105 |
|  | I-squared = 0.0% | I-squared =0.0% | I-squared =50.55% |
